# Strategies for the Isolation and Identification of Gastric Cancer Stem Cells

**DOI:** 10.1155/2024/5553852

**Published:** 2024-06-06

**Authors:** Jianhua Wang, Jie Qu, Qiang Hou, Xueping Huo, Xiangrong Zhao, Le Chang, Cuixiang Xu

**Affiliations:** ^1^ Shaanxi Provincial Key Laboratory of Infection and Immune Diseases Shaanxi Provincial People's Hospital, Xi'an 710068, China; ^2^ Second Department of General Surgery Shaanxi Provincial People's Hospital, Xi'an 710068 710068, China; ^3^ Department of Graduate School Yan'an University, Yan'an 716009, China; ^4^ Shaanxi Engineering Research Center of Cell Immunology Shaanxi Provincial People's Hospital, Xi'an 710068, China

## Abstract

Gastric cancer stem cells (GCSCs) originate from both gastric adult stem cells and bone marrow cells and are conspicuously present within the histological milieu of gastric cancer tissue. GCSCs play pivotal and multifaceted roles in the initiation, progression, and recurrence of gastric cancer. Hence, the characterization of GCSCs not only facilitates precise target identification for prospective therapeutic interventions in gastric cancer but also has significant implications for targeted therapy and the prognosis of gastric cancer. The prevailing techniques for GCSC purification involve their isolation using surface-specific cell markers, such as those identified by flow cytometry and immunomagnetic bead sorting techniques. In addition, *in vitro* culture and side-population cell sorting are integral methods in this context. This review discusses the surface biomarkers, isolation techniques, and identification methods of GCSCs, as well as their role in the treatment of gastric cancer.

## 1. Introduction

Recent data from the World Health Organization indicate that gastric cancer is the fourth leading cause of cancer-related mortality, particularly in East Asia [1]. Despite advancements in surgical interventions and treatments, individuals diagnosed with gastric cancer have a limited 5-year survival rate and often succumb to metastatic progression [2]. To improve the prognosis of patients with gastric cancer, it is imperative to explore novel therapeutic modalities.

In recent years, advancements in cancer stem cell (CSC) research have contributed significantly to the refinement of cancer treatments. A century ago, European pathologists made the seminal discovery that tumors consist of a heterogeneous assembly of partially differentiated cell types [3]. It was not until 1994 that the presence of CSCs was initially demonstrated in human acute myeloid leukemia [4]. Since that milestone, stem cells have been identified across various tumor types, and researchers have employed available data to formulate a consensus definition of CSCs as “a cellular population within a tumor endowed with the capacity for self-renewal, yielding a diverse spectrum of cancer cells, and exhibiting tumorigenic potential” [5]. CSCs constitute a distinct subset within neoplastic cell populations and are acknowledged as pivotal contributors to tumorigenesis and progression, particularly in aggressive heterogeneous tumors. Tumor cells characterized by high proliferative potential are effectively eradicated during therapeutic interventions, such as chemotherapy or radiotherapy. However, CSCs exhibit resistance to pharmacological agents and persist even after treatment. This persistence contributes to alterations in the tumor, which may facilitate tumor recurrence. Hence, the cornerstone of effective tumor management is the precise targeting and timely elimination of these high-risk cells. This strategy not only hinders tumor progression but also alleviates the likelihood of tumor recurrence [6].

Research pertaining to gastric cancer stem cells (GCSCs) has emerged as a focal point in gastric cancer treatment. The origin, isolation methods, surface labeling techniques, and associated signaling pathways of GCSCs are areas of current research. Potential reservoirs of GCSCs include mutant gastric stem cells and bone marrow-derived cells [7]. Gastric stem cells are adult pluripotent stem cells inherent in gastric tissue that can differentiate into diverse types of gastric mucosal cells. Upon oncogenic mutation, these initially benign gastric stem cells undergo a transformative process, culminating in the acquisition of a neoplastic phenotype, leading to atypical hyperplasia of the gastric mucosa and the subsequent formation of gastric cancer [8]. Bone marrow-derived cells fuse with local gastric epithelial cells, resulting in tissue remodeling, transformation, and the progression of malignancy [9]. Bone marrow-derived cells have been identified in studies utilizing a mouse model of *Helicobacter*-induced gastric cancer; these cells exhibit enhanced plasticity and a proclivity to migrate through peripheral organs, potentially influenced by inflammation and tissue damage [10]. The methods for isolating GCSCs can be broadly categorized into two primary approaches. The first entails sorting based on the surface biomarkers of GCSCs. The second approach involves sorting methods specifically tailored to the characteristics of stem cells, encompassing techniques such as serum-free cultivation and drug-resistant isolation [11]. Understanding the various signaling pathways involved in the proliferation and differentiation of GCSCs can help us better understand their roles in gastric cancer development. The most common signaling pathways include the WNT, NOTCH, Hedgehog, HIPPO, and PI3K/AKT/mTOR signaling pathways [10, 12, 13]. Currently, research on the targets of GCSCs mainly involves several related genes within popular signaling pathways, such as Her2, EGFR, and PI3K. In 2014, The Cancer Genome Atlas initiative further characterized the molecular features of gastric cancer and proposed four molecular subtypes: Epstein–Barr virus-positive, microsatellite unstable, genome stable, and chromosomal instability types [14]. The molecular typing of The Cancer Genome Atlas helps in the selection of targeted drugs for patients with gastric cancer. For example, for Epstein–Barr virus- and microsatellite unstable-type tumors, the treatment strategy may include PI3K inhibitors [15]. The identification of biomarkers and molecular classifications has provided important clues for improving early diagnosis and therapeutic interventions for rare gastric cancer types with unique histopathological characteristics.

GCSCs play important roles in tumorigenesis and development, epithelial–mesenchymal transition, metastasis, recurrence, and prognosis. Given that GCSC research is an emerging field and that many details are not yet known, many issues need to be addressed for future advancements. This review provides a comprehensive summary of the characteristics, isolation techniques, and identification methods for GCSCs. In addition, the potential applications of surface markers associated with GCSCs in cancer diagnosis and treatment were investigated. The overarching goal of this review is to present novel research perspectives and offer potential solutions to advance the diagnosis and treatment of gastric cancer.

## 2. Biomarkers and Targeted Therapeutic Strategies for GCSCs

**Figure 1:**
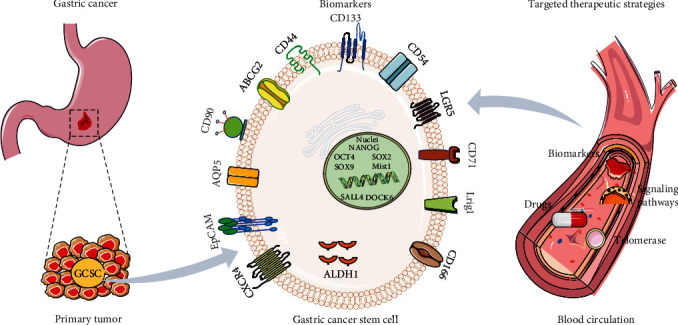
Biomarkers and therapeutic strategies for gastric cancer stem cells (GCSCs). Various stem cell biomarkers associated with gastric cancer, including CD44 [16] and CD133 [21], have been identified, primarily localized to the cellular membrane surface. In the realm of gastric cancer management, strategic immunotherapeutic strategies and the development of small molecule inhibitors have been intricately crafted to specifically target GCSCs.

To date, various CSCs have been shown to exhibit distinct markers. Among these, CD44 and CD133 are the most frequently used cell surface markers for CSC identification. In recent years, attention has been directed toward investigating the cell markers associated with GCSCs. In 2007, Takaishi et al. [16] reported that gastric cancer cells exhibited heightened self-renewal and differentiation potential within the CD44+ subgroup, which is consistent with the phenotypic characteristics of GCSCs. Recent studies have identified AQP5 as a novel specific surface marker of GCSCs. Functionally, AQP5 facilitates sphere formation, proliferation, migration, and invasion of gastric cancer cells *in vitro*. Moreover, AQP5 collaborates and synergizes with LGR5 to promote tumorigenesis in GCSCs [17]. Numerous molecular markers have also been associated with GCSCs (Figure 1 and Table 1). Cell membrane surface markers, such as CD133 (a human glycoprotein encoded by the PROM1 gene) [21], Lgr5 (a G-protein-coupled receptor rich in leucine repeats) [22], and EpCAM (an epithelial cell adhesion factor) [25], predominantly contribute to the development of gastric cancer. CD44 (a cell surface transmembrane glycoprotein) [39] and ABCG2 (adenosine triphosphatase binding cassette transporter G superfamily member) [40] are primarily implicated in the prognosis of gastric cancer. CD24 (a glycosylphosphatidylinositol-anchored membrane protein) [25] and ALDH1 (acetaldehyde dehydrogenase) [38] have the potential to enhance tissue resistance in gastric cancer. Furthermore, SOX2 (the sex-determining region Y box protein) exerts a significant impact on the metastasis and invasion of gastric cancer [34]. GCSCs are implicated in chemotherapy resistance, tumor invasion, and metastasis, thus representing a potential direction for the future treatment of gastric cancer. Traditional treatments cannot cure the tumor fundamentally and are limited by the possibility of metastasis and recurrence, while treatments targeting tumor stem cells may treat the tumor fundamentally [41]. Currently, there are several targeted therapeutic strategies for GCSCs: (1) targeted therapy against specific markers on the surface of GCSCs, which distinguish them from other cells, leading to the effective killing of tumor stem cells [42]. (2) Targeted therapy for GCSC signaling pathways, since aberrant regulation of these pathways leads to abnormal proliferation and differentiation [43]. (3) Targeted therapy against telomerase in GCSCs, which have higher telomerase activity and shorter telomere length than normal cells, can induce senescence and apoptosis, halting the tumor stem cell cycle with minimal damage to normal cells [44]. (4) Stemness-targeting drugs for gastric cancer treatment to combat multidrug resistance in tumor stem cells: utilizing novel drugs to target naturally resistant CSCs has the potential to reduce cancer recurrence rates and improve the efficacy of tumor therapy. Zhang et al. [45] demonstrated that PIN1 suppresses the chemotherapy resistance of gastric cancer cells by targeting GCSCs and multiple signaling molecules and biomarkers in gastric cancer. Similarly, targeted inhibition of the RAC1 pathway prevented the epithelial–mesenchymal transition and CSC phenotypes in gastric adenocarcinoma, which in turn inhibited drug resistance [46]. Researchers have discovered many drugs that target the stemness of gastric cancer, including marketed chemotherapy drugs, clinical drugs for other diseases, small-molecule drugs, and traditional Chinese medicines [6]. Cao et al. [47] found that apatinib inhibits the stemness of gastric cancer cells through the Hedgehog signaling pathway. Nguyen et al. [48] found that all-trans retinoic acid targets GCSCs and inhibits patient-derived gastric carcinoma tumor growth. All-trans retinoic acid downregulates the expression of CSC markers, CD44 and ALDH (aldehyde dehydrogenase), and stemness genes, such as Klf4 and Sox2, and induces tumor sphere differentiation [48]. Traditional Chinese medicines have also been reported to inhibit the stemness of gastric cancer cells. Li et al. [49] found that Sijunzi decoction inhibited the stemness of gastric cancer cells by inhibiting the transcriptional activity of *β*-catenin. It is believed that some of the above drugs will enter clinical trials and be approved by the FDA in the near future to benefit patients with gastric cancer.

## 3. Methods for Isolating GCSCs

### 3.1. Isolation of GCSCs by Serum-Free Suspension Culture *In Vitro*

**Figure 2:**
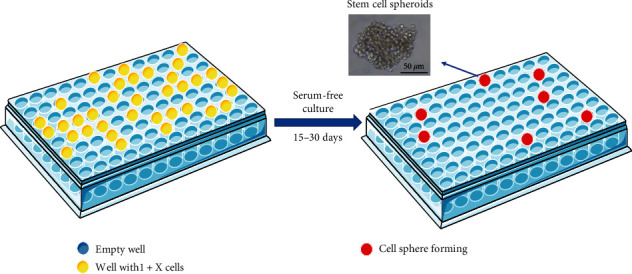
Isolation of gastric cancer stem cells (GCSCs) via serum-free suspension culture *in vitro*. The method of infinite dilution induces a phenotypic transition in GCSCs, leading to the acquisition of a spherical morphology, while normal gastric cancer cells undergo apoptosis.

The *in vitro* serum-free suspension culture method involves the supplementation of serum-free media with diverse growth factors, such as EGF and bFGF, which serve as substitutes for serum. This serum-free medium preserves critical nutrients upon which cells rely, such as insulin, transferrin, and progesterone. Simultaneously, it eliminates the prodifferentiation agents present in the serum. Consequently, cells exhibiting multidirectional differentiation potential can be sustained and developed into spheres. In contrast, regular cells tend to undergo gradual cell death when exposed to serum-free medium, which facilitates their isolation [50]. In most studies focusing on isolating GCSCs, targeted cells are typically inoculated into serum-free, low-adhesion porous plates to facilitate the formation of cell spheres (Figure 2). The *in vitro* culture isolation method serves as a preliminary screening approach for tumor stem cells and is notably advantageous because of its independence from specific surface markers. Although this method has been extensively applied for the isolation and cultivation of various tumor stem cells, it has certain drawbacks. Specifically, it can only achieve the initial isolation of tumor stem cells, which is characterized by a diminished separation efficiency and an extended culture cycle [51–53]. Recently, several new *in vitro* culture methods have been successfully developed, including pellet culture [54], organoids [55], liquid overlay [56], hanging drop, and microfluidics [57]. Compared to the traditional *in vitro* culture of tumor stem cells, 3D tumor stem cell culture exhibits the capability to release elevated levels of cytokines and chemokines. This modality significantly influences cell proliferation, vitality, and migration and promotes angiogenesis [58]. Lee et al. [59] successfully isolated individual cell spheres representing GCSCs by employing a hybrid approach that combined a serum-free culture-limited dilution method with a microtiter-based spheroid culture plate protocol. The resulting spheres exhibited a round morphology with distinct and tightly bound characteristics. Notably, these isolated cell spheres demonstrated significantly elevated expression of genes associated with stemness and displayed enhanced antiapoptotic properties compared to those obtained using conventional culture methods [59]. Consequently, the organoid culture technique holds immense potential in GCSC research.

### 3.2. Isolation of GCSCs by Fluorescence-Activated Cell Sorting (FACS)

**Figure 3:**
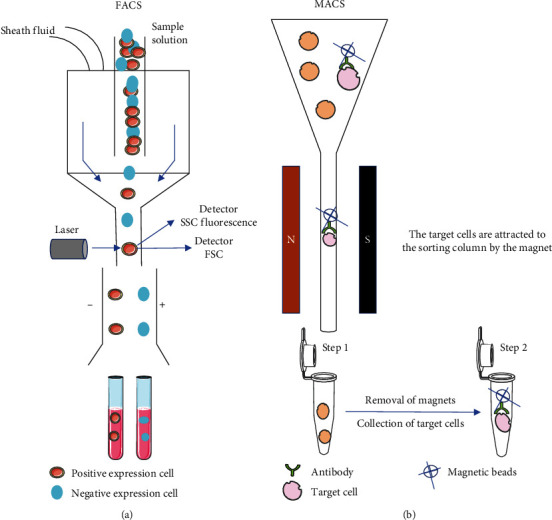
Isolation of gastric cancer stem cells (GCSCs) via fluorescence-activated cell sorting (FACS) and magnetic-activated cell sorting (MACS) methods. The isolation of GCSCs based on surface markers is a fundamental requirement for both FACS (a) and MACS (b) procedures. FACS and MACS represent distinct methodologies distinguished by their respective underlying mechanisms. FACS relies on immunofluorescence, whereas MACS utilizes magnetic fields and magnetic nanoparticles.

Originally proposed in 1972, FACS utilizes a flow cytometry-based technique to sort cell samples labeled with fluorescein-coupled antibodies (Figure 3(a)). It distinguishes between target and nontarget cells using a fluorescent system. Flow cytometry assesses the physical and chemical characteristics of cells by analyzing the scattered light and fluorescence signals emitted by the cells in the flow upon laser irradiation. Considered the “gold standard” for cell sorting, flow cytometry ensures high-purity cell isolation with a high recovery rate, while maintaining a fully enclosed operating environment to minimize contamination risks. However, this method requires expensive equipment, is time-consuming, and can significantly affect the cell stimulation and activity of sorted cells. In addition, it is limited to sorting one cell sample at a time, rendering it impractical to sort target cells from different samples [60]. Takaishi et al. [16] employed CD44 as a surface marker to identify GCSCs and effectively utilized FACS to isolate CD44+ cells. Subsequently, the authors rigorously validated the stem cell properties of the isolated subpopulation [16]. In another study, Zhang et al. [61] directly isolated the CD44+ CD24+ gastric cancer cell subset from AGS gastric cancer cells using flow-sorting technology. Discerning analysis of this specific cellular subset revealed heightened self-renewal and differentiation capacities concomitant with robust tumorigenic potential [61]. Using a divergent approach, Pádua et al. [62] implemented a SOX 2/OCT 4 activity-based traceability reporting system (SO6-GFP) to selectively isolate stem-like cells from human gastric cancer cell lines. Employing FACS, the researchers systematically categorized gastric cancer cells into distinct SO6+ and SO6− cell populations. The results of this meticulous sorting process revealed that SO6+ cells exhibited unequivocal stem cell characteristics and augmented resistance to 5-fluorouracil treatment [62]. With the discovery of an expanding array of stem cell surface markers, FACS will become increasingly important for sorting GCSCs.

### 3.3. Isolation of GCSCs by Magnetic-Activated Cell Sorting (MACS)

MACS is based on the fundamental principle of utilizing monoclonal antibodies targeting unlabeled antigens and proteins as primary antibodies. The specific antibody is incubated with a single-cell suspension under hydrodynamic conditions, followed by the addition of a secondary antibody labeled with immunomagnetic beads. Upon exposure to a magnetic field generated by a specialized permanent magnet, the labeled cell suspension is adsorbed onto a magnetic sorting column. Subsequently, the magnetic field is withdrawn, facilitating elution of the adsorbed cells from the column (Figure 3(b)). This process achieves efficient separation and collection of stem cells [63]. Using MACS, GCSCs were isolated from cultured gastric cancer cells, with CD133 and CD166 serving as surface markers. Verification by flow cytometry indicated that the proportion of isolated CD133+/CD166+ GCSCs surpassed 90% [43]. Yoon et al. [64] used MACS to selectively isolate a CD44+ subpopulation of gastric cancer cells. This isolated subpopulation exhibited distinct migratory and invasive characteristics, along with notable resistance to both 5-fluorouracil and cisplatin [64]. The merits of MACS for tumor stem cell sorting include (1) straightforward equipment and cost-effectiveness, (2) safety and nonstimulation, preserving cell activity and function, and (3) the capacity to obtain a sorted cell population with high purity (90%–99%) and substantial recovery rates. However, one limitation of this method is its prolonged operational steps and extended sorting time compared with FACS.

### 3.4. Isolation of GCSCs by the Side Population (SP) Method

**Figure 4:**
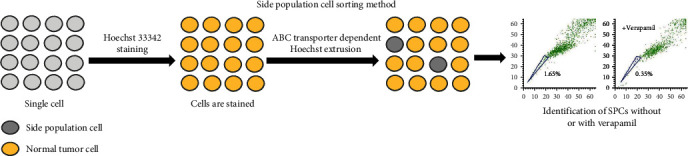
Isolation of gastric cancer stem cells (GCSCs) by the side population (SP) method. The Hoechst SP technique is a flow cytometry-based approach devised for the isolation of stem cells, leveraging the dye efflux characteristics of ATP-binding cassette (ABC) transporters. Notably, the fluorescent DNA-binding dye Hoechst 33342 is actively extruded by ABC transporters and preferentially accumulates in stem cells.

Hoechst 33342 is an exclusion dye widely used for separating SP and non-SP cells in cell lines and tissues. When cells are stained with Hoechst 33342, some of the cells appear “beak-like” or “trailing” in flow cytometry scattering analysis, and these cells are able to pump the dye out of the cell rapidly, leaving the cells with low levels of intracellular Hoechst 33342; these cells are known as SP cells [65]. The main reason for this ability of SP cells is that their membrane surface highly expresses the ATP-binding cassette transporter superfamily, including multidrug resistance protein 1 (MDR1) and ABCG2. The function of these transporter proteins can be inhibited by verapamil, such that the SP cell population is significantly reduced or even disappears when verapamil is added (Figure 4) [66]. It has been continuously demonstrated that SP cells have similar properties to stem cells, such as self-renewal, multidirectional differentiation, drug resistance potential, and high tumorigenicity [67]. Researchers have confirmed the presence of SP cells in human gastric cancer cell lines. Furthermore, SP cells exhibit clonal growth capacity and self-renewal properties when cultured in conditioned medium [68]. The cell population selected using this method exhibits increased proliferation, drug resistance, and elevated expression of stem cell surface markers. However, it is imperative to note that the identification of SP cells using this technique merely implies the presence of a certain proportion of stem cells within the SP and cannot definitively establish the SP cells as tumor stem cells. Consequently, this screening method lacks stringent criteria typically associated with stem cell identification [69]. Zhang et al. [70] demonstrated that SP cells isolated from the gastric cancer cell line BGC-823 did not uniformly exhibit CSC characteristics. This suggests that not all SP cells derived from gastric cancer cell lines harbor stem-like properties [70]. Moreover, the cells underwent damage and contamination following dye treatment and flow cytometry sorting. Consequently, the limited number of collected cells hinders effective long-term regenerative cell culture, compromising subsequent experimental implementation and overall execution.

### 3.5. Isolation of GCSCs Using the Aldefluor Assay Method

The aldefluor assay is used to isolate tumor stem cells based on ALDH activity. Previous investigations have revealed higher expression of ALDH in GCSCs than in normal cancer cells. Consequently, this approach is instrumental in the isolation of GCSCs [71]. ALDH activity is assessed through the cleavage of a fluorescent substrate (BODIPY-aminoacetaldehyde or BAAA) consisting of an aminoacetaldehyde moiety linked to the BODIPY fluorochrome. To precisely quantify cell numbers exhibiting elevated ALDH activity, a pan-ALDH inhibitor, diethylaminobenzaldehyde, is employed as a control [11]. Cells expressing increased levels of ALDH and demonstrating increased activity exhibit greater fluorescence intensity, facilitating their discernment by flow cytometry. Researchers have effectively applied aldefluor analysis to isolate ALDH-bright cells from two human gastric cancer cell lines: MKN-45 and SGC-790. These isolated cells exhibited enhanced self-renewal and differentiation capabilities, indicating their stem cell characteristics [72]. Unlike other methods of sorting stem cells using GCSC markers, the aldefluor assay measures internal cellular enzyme activity to provide a novel alternative.

### 3.6. Isolation of GCSCs by the Chemotherapy Induction Screening Method

**Figure 5:**
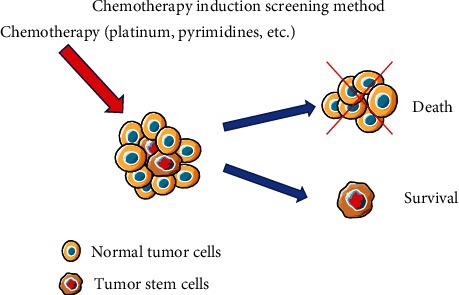
Isolation of gastric cancer stem cells (GCSCs) utilizing the chemotherapy induction screening method. Leveraging the inherent drug resistance of stem cells, GCSCs can be initially isolated through exposure to chemotherapeutic agents.

Heterogeneity within tumors manifests as the diverse responses of tumor cells to chemotherapy drugs. Certain cells may succumb to treatment, whereas others persist, contributing to disease progression [73]. Tumor stem cells, characterized by unique functional and molecular features, such as dysregulated apoptosis/survival pathways and interactions with the microenvironment, may exhibit therapeutic sensitivity, leading to the induction of cell resistance [74]. In the context of gastric cancer, stem cells have demonstrated robust resistance to chemotherapy, prompting their isolation using a chemotherapy induction screening method (Figure 5). In a study conducted by Xu et al. [75], the human gastric cancer cell lines SGC7901 and AGS were subjected to 5-fluorouracil exposure, revealing that surviving gastric cancer cells exhibited resistance to this chemotherapeutic agent. Notably, these resistant cells displayed stem cell characteristics, including a high proportion of quiescent cells, augmented self-renewal capacity, and enhanced tumorigenicity. Additionally, resistant cells were enriched with cells expressing CD133+, CD326+, and CD44+ CD24− [75]. Furthermore, upon treatment of SGC7901 gastric cancer cells with 1 mg/mL vincristine for 72 hr, the treated cells displayed mesenchymal characteristics, as evidenced by the upregulation of the mesenchymal markers snail, twist, and wave protein. Comparative analysis with untreated cells revealed the ability of treated cells to undergo significant asymmetrical division, demonstrating their self-renewal capacity. Importantly, these cells exhibited multidrug resistance and substantial *in vivo* tumorigenic properties [76]. The successful application of the chemotherapy induction screening method in studying other tumor stem cells underscores its potential specificity, warranting further exploration of its applicability in the classification of GCSCs.

### 3.7. Isolation of GCSCs by the Large Retinal Milk Spot Screening Method

Primordial lymphoid tissues known as milk spots are predominantly situated within the peritoneal cavities of humans and animals, specifically in the large omentum, mesentery, and pelvic floor. They serve as implantation sites for malignant cells and may contribute to peritoneal dissemination [77]. The large omental papilla consists of large aggregates of macrophages and lymphocytes, which are mainly involved in removing tumor cells and bacteria [78], and is a common site of metastasis in gastric cancer. Taking advantage of this property of large omental plaques, Cao et al. [79] combined the tumor stem cell theory and suggested that GCSCs infiltrated the plaques and then proliferated and differentiated into gastric cancer progenitor cells, which is the basis of gastric cancer micrometastasis formation. In this context, the large omental plaque becomes an efficient “natural filter” for screening GCSCs. By collecting progenitor cells from breast spot micrometastases, individual gastric cancer cells were isolated and transplanted into NOD/SCID mice. The validation results showed that the gastric cancer cells collected using this method were more tumorigenic, with significantly higher expression of CD133 and CD324 than gastric cancer cells collected without screening. With a simpler method of isolating and purifying GCSCs, this novel stem cell isolation model is crucial for screening and maintaining the development of GCSCs. This will also aid in the discovery of potential markers of GCSCs, which will be a significant advancement in the study of these cells.

### 3.8. Application of Organoid Technology in the Precision Treatment of Gastric Cancer

In recent years, organoid technology has been applied to understand the biology of tumor stem cells. Organoid technology facilitates the effective cultivation of tumor cells by emulating their *in vivo* microenvironment. Tumor-like organoids exhibit a high degree of fidelity, preserving the histocellular morphology, genomic features, and drug sensitivity characteristics of the original tumor [80, 81]. Patient-derived organoids (PDOs) are 3D primary cultures that can be maintained and propagated *in vitro* while maintaining the same molecular and phenotypic features as the original tumor. PDOs closely mimic the characteristics of tumor tissues and preserve the heterogeneity of individual tumors. They can be used for functional analyses, such as high-throughput drug screening, disease modeling, and new drug development, providing a unique platform for researchers and clinicians to study the biology of tumors, test the efficacy of drugs, and develop personalized therapeutic strategies for specific patients [82]. In a study by Seidlitz et al. [83], 20 PDO lines were established from patients with gastric cancer, and four cultures were selected for further studies because of their high proliferation rates. These cultures were treated with 5-fluorouracil, oxaliplatin, irinotecan, epirubicin, and docetaxel, which are standard and commonly used clinical drugs. Significantly different responses were observed for the four PDOs [83]. Yan et al. [84] established a biobank that included 17 normal and 46 gastric cancer organoid lines from 34 patients. They demonstrated that the organoids replicated the genomic and gene expression signatures of the original tumor and identified potential targets for personalized therapy, including napabucasin, abemaciclib, and the ATR inhibitor VE-822 [84]. Another important application of organoids is the possibility of employing them to study the mechanisms of resistance to both conventional and experimental therapies [85]. Consequently, the organoid culture technique holds immense potential in GCSC research.

### 3.9. Isolation of GCSCs by Multiple Sorting Methods

It is important to note that individual stem cell isolation methods have limitations. Researchers usually isolate GCSCs not by a single method but by combining two or more isolation methods to obtain a larger number of GCSCs with higher purity. For example, when the serum-free suspension culture method is used alone, although a larger number of tumor cell spheres can be obtained, the cycle time is long, and the purity of the stem cells is not guaranteed. When FACS is applied alone, although GCSCs containing specific surface markers can be accurately isolated, the cell numbers are small, and the cell status is poor. Therefore, many researchers have combined the serum-free culture method and flow sorting technology to screen gastric cancer cells using flow sorting technology and have further screened and expanded the culture of the obtained tumor stem cells using serum-free culture technology, which has greatly improved the quantity and purity of the obtained GCSCs [28, 61]. The amalgamation of multiple isolation methods mitigates the drawbacks inherent in each individual method. This comprehensive strategy ensures that the final quantity and purity of the isolated GCSCs meet the requisite standards for experimental purposes, thereby providing a robust foundation for further research on GCSCs. The adoption of a combined approach encompassing various isolation methods is an emerging trend in the investigation of isolated GCSCs. The advantages and disadvantages of common methods for isolating GCSCs are shown in Table 2.

## 4. Identification of GCSCs

### 4.1. Identification of the Tumorigenic Capacity of Stem Cells

The defining characteristic of all stem cells is their dual capacity for self-renewal and differentiation. Analogous to their normal counterparts, the assessment of tumor stem cell activity hinges on the scrutiny of their self-renewal capabilities and the potential for sustained proliferation. The most reliable evaluation of these criteria requires the use of animal models, with tumorigenicity emerging as a paramount metric for the identification of stem cells. Empirical evidence indicates that nearly all transplanted mice manifest tumorigenicity when inoculated with a mere 100 tumor stem cells, whereas approximately 10^5^ untreated normal tumor cells are required to achieve comparable outcomes. Significantly larger cell quantities are required when employing nonstem cells in tumorigenicity assays, underscoring the discriminatory potential of this method [86]. The principle of this methodology involves grafting the cell population under investigation into *in vivo* sites in immunodeficient mice, typically the NOD/SCID strain. To ensure a pathogen-free environment, mice receive sterile provisions encompassing food, water, and cages. Tumor development, measured using parameters such as volume, mass, and growth rate, is then systematically monitored at distinct intervals until an ethical endpoint is reached. The ethical endpoint, characterized by indicators such as respiratory distress, erect hair, anemia, or weight reduction exceeding 10% of the initial body weight, signifies the termination of the experiment [28]. Importantly, the execution of animal experiments necessitates compliance with the Animal Experimentation Ethics Committee of the Center, emphasizing a commitment to minimizing the number of animals utilized in experiments and mitigating the associated distress.

### 4.2. Identification of Stem Cell Functionality

GCSCs exhibit enhanced capabilities for self-renewal, cell differentiation, cell proliferation, resistance to chemotherapy drugs, migration, and invasion compared to conventional gastric cancer cells. Therefore, these functional properties can be used to identify GCSCs. The self-renewal capacity of individual cells in serum-free medium is assessed through the quantification of cell sphere formation, which is typically measured as the cell sphere formation efficiency. This criterion stems from the unique ability of stem cells to differentiate into diverse phenotypes and generate cell spheres in the presence of growth factors [87]. The CCK8 assay is simple, and the results are intuitive enough to become the main method for stem cell proliferation detection [11]. The principle is that the WST-8 in the reagent can be reduced by mitochondrial deoxygenase to a water-soluble methanogen, and the faster the cells proliferate, the darker the color. A growth curve can be plotted by measuring the absorbance at 450 nm for several consecutive days, which visually reflects cell proliferation. The primary approach for assessing chemotherapy resistance involves the inoculation of cells in 96-well plates, followed by the addition of common gastric cancer chemotherapy drugs, such as cisplatin and vincristine, after 24 hr. Subsequently, the MTT method is employed to ascertain cell activity after coculturing for a specific duration at the IC50 concentration [87]. The determination of drug resistance in GCSCs typically relies on the ratio of the number of viable cells in the drug treatment group to that in the control group [88, 89]. To further investigate the migratory and invasive capabilities of sorted GCSCs and normal gastric cancer cells, a transwell experiment can be conducted to compare the number of cells traversing the chamber under identical conditions. Notably, the ability of GCSCs to form colonies, migrate, and invade is markedly greater than that of conventional gastric cancer cells [90].

## 5. Summary and Outlook

Increasing evidence underscores the dual role of GCSCs in both instigating and propelling the progression of gastric cancer, while also playing a pivotal role in its recurrence. Recent studies on GCSCs have yielded significant results, leading to the discovery and utilization of an expanding array of GCSC surface markers in scientific investigations. Currently, MACS and FACS are the most reliable techniques for CSC isolation, despite inherent technical challenges. Notably, FACS entails cell loss due to stress during the sorting process, thereby diminishing sorting efficiency [91]. MACS encounters challenges related to nanoparticle bead endocytotoxicity, postseparation bead shedding, and concurrent separation of multiple markers [92]. Beyond conventional methodologies, novel approaches, such as laser microdissection and microfluidics, are emerging for the isolation of tumor stem cells. Laser capture microdissection affords precise control over target regions containing cells of interest through a laser dissection method [93]. Laser capture microdissection is an excellent tool for isolating tissue areas or specific cell populations within a tissue without contamination by surrounding cells. However, this technique has not been used to isolate GCSCs. Microfluidics, operating on a microscopic scale, serves as a technical platform for fluid and particle transport, and its main application is the isolation of circulating tumor cells [57]. The integration of microfluidics technology with other techniques, such as immunoamplification electrophoresis and immunofluorescence, enhances the efficiency of tumor stem cell sorting [94]. Although these methods are not currently used to isolate GCSCs, they could be used in the future as the technology continues to evolve and further research on GCSCs is conducted. With the continuous development of tumor stem cell sorting methods, efficient sorting of GCSCs is of great significance. The study of GCSCs provides new insights into the molecular mechanisms and pathways governing gastric cancer cell regulation. This research not only advances our understanding but also contributes to breakthroughs in the diagnosis and treatment of gastric cancer, laying the groundwork for targeted therapies in this field.

## Figures and Tables

**Table 1:** Main cell-surface/intracellular markers of gastric cancer stem cells.

Marker	Location	Function	Functions associated with gastric cancer	References
CD44	Membrane	Hyaluronic acid receptor	Involvement in gastric cancer metastasis, invasion; associated with drug resistance	[16]
CD44V	Membrane	Hyaluronic acid receptor	Involvement in gastric cancer metastasis, invasion; associated with drug resistance	[18, 19]
CD90	Membrane	Immunoglobulin superfamily/Thy-1 cell-surface antigen	Involvement in gastric carcinogenesis and metastasis	[20]
CD133	Membrane	Transmembrane glycoprotein	Accelerate the development of gastric cancer; associated with drug resistance	[21, 22]
AQP5	Membrane	Recombinant aquaporin 5	Participation in self-renewal and development of gastric cancer	[17]
Lgr5	Membrane	Leucine-rich repeat-containing G-protein coupled receptor 5	Intestinal epithelial metaplasia involved in gastric carcinogenesis; promote the development of gastric cancer	[17]
Lrig1	Membrane	Regulatory factor of cell cycle	Involved in cell-cycle repression and response to oxidative damage	[23]
ABCB1 ABCG2	Membrane	ABC transporters	Associated with drug resistance	[24]
CD54	Membrane	Cell adhesion molecule	Involvement in gastric cancer metastasis, invasion	[25]
EpCAM	Membrane	Epithelial cell adhesion molecule	Involved in gastric cancer development, progression, and lymph node metastasis	[26]
CD71^−^	Membrane	Transferrin receptor	Involvement in gastric carcinogenesis, invasion	[27]
CXCR4	Membrane	C-X-C chemokine receptor type 4	Involvement in peritoneal metastasis of gastric cancer; associated with drug resistance	[28]
CD24	Membrane	Cell adhesion molecules	Involvement in gastric cancer growth, invasion, and metastasis	[29]
CD166	Membrane	Cell adhesion molecule	Involvement in gastric cancer growth, invasion, and metastasis	[30]
Mist1	Nucleus	Transcriptional factor	Involvement in gastric cancer growth and metastasis	[31]
DOCK6	Nucleus	Dock-C subfamily guanine nucleotide exchanger	Associated with drug resistance	[32]
NANOG	Nucleus	Transcription factor	Involvement in gastric cancer growth and metastasis	[33]
Sox2	Nucleus	Sex determining region Y-box 2	Involved in proliferation and metastasis of gastric cancer; associated with drug resistance	[34]
Sox9	Nucleus	The HMG box transcription factor	Intestinal epithelial metaplasia involved in gastric carcinogenesis; promote the development of gastric cancer	[35]
SALL4	Nucleus	Zinc-finger transcription factor	Involvement in gastric carcinogenesis and metastasis	[36]
ASPM	Cytoplasm nucleus	Abnormal spindle-like microcephaly-associated protein	Involvement in gastric carcinogenesis	[37]
ALDH1	Cytoplasm	Aldehyde dehydrogenase 1	Involved in the development and progression of gastric cancer; associated with drug resistance	[38]

**Table 2:** Advantages and disadvantages of methods for isolating gastric cancer stem cells.

Projects	Advantages	Disadvantages
*In vitro* culture sorting method	No specific surface markers required	Primary sorting and long cell culture cycle
Fluorescence-activated cell sorting method (FACS)	High precision and speed; simultaneous sorting of multiple surface markers	Need for specific surface markers; possible damage to cells
Magnetic-activated cell sorting (MACS)	Easy to operate; no damage to cells	Need for specific surface markers; one surface marker at a time sorting
Side group cell sorting method	No specific surface markers required	Primary sorting and some toxic effects on cells
Chemotherapy induction screening method	Easy to operate; no specific surface markers required	Primary sorting
Large retinal milk spot screening method	Highly efficient “natural filter” for screening gastric cancer stem cells	Currently less used

## Data Availability

In this review, we only summarized the existing methods for isolating and identifying gastric cancer stem cells and did not produce new data.
